# Two potentially lethal conditions of probable immune origin occurring in a pregnant woman: a case report

**DOI:** 10.1186/s13256-018-1701-4

**Published:** 2018-06-06

**Authors:** H. M. Senanayake, M. Patabendige

**Affiliations:** 1 Faculty of Medicine, University of Colombo, Colombo, Sri Lanka; 2 University Obstetrics Unit, De Soysa Hospital for Women, Colombo, Sri Lanka

**Keywords:** Thrombotic thrombocytopenic purpura, Pregnancy, Peripartum cardiomyopathy, Autoimmunity, Immune hyperreactivity

## Abstract

**Background:**

Thrombotic thrombocytopenic purpura and peripartum cardiomyopathy are potentially lethal complications of pregnancy. We describe a case in which both of these developed in the same patient. The etiologies of both conditions remain uncertain, but they share immune hyperreactivity as a possible cause.

**Case presentation:**

A 33-year-old Lankan primigravida gave birth at 38 weeks of gestation by cesarean section when she presented with right-sided abdominal pain and a provisional diagnosis of appendicitis. Her pain persisted postoperatively, and on the second postoperative day, she physicaly collapsed suddenly with abdominal distention. Immediate laparotomy revealed generalized oozing from the peritoneum resulting in hemoperitoneum and intestinal hemorrhage. Her laboratory reports showed microangiopathic hemolytic anemia and thrombocytopenia. She also had elevated liver enzyme, lactate dehydrogenase, and creatinine concentrations. A diagnosis of thrombotic thrombocytopenic purpura was made. After a steady recovery, she was discharged from the hospital on the 16th postoperative day, but 12 hours later, she was readmitted with acute-onset progressively worsening shortness of breath. Echocardiography confirmed peripartum cardiomyopathy. She was treated with a bromocriptine and heart failure regimen. At 6 weeks postpartum, her laboratory test results and cardiac function had improved.

**Conclusions:**

A possible autoimmune association might have caused both conditions in our patient. This case report serves as a warning message that pregnant women with one possible condition with autoimmune association could go on to develop other similar conditions.

## Background

Thrombotic thrombocytopenic purpura (TTP) and peripartum cardiomyopathy (PPCM) are rare, potentially lethal complications of pregnancy. We report a case in which both of these conditions developed in the same patient. The etiologies of both of these entities are uncertain, but they share immune hyperreactivity as a possible cause.

## Case presentation

A 33-year-old Lankan primigravida was admitted at 38 weeks of gestation with episodic right-sided abdominal pain. Her pain was gradually worsening with no change in urinary and bowel habits. Her past medical, surgical, family, and psychosocial histories were unremarkable. A cesarean delivery was performed with a provisional diagnosis of appendicitis because no other explanation could be found for the pain. A preoperative platelet count of 130 × 10^3^/L was noted, but it was not investigated further. The delivery was concluded uneventfully with a delivery of a nonasphyxiated baby girl weighing 2.8 kg. The patient’s appendix was noted to be normal. Her pain persisted postoperatively, and on the second postoperative day, she physicaly collapsed suddenly with abdominal distention. An episode of hematemesis followed shortly afterward. Her hemoglobin level was 5 g/dl, and her platelet count was 60 × 10^3^/L. Intraperitoneal and intestinal bleeding was diagnosed, and an emergency laparotomy was performed. Her conjunctivae were noted to be icteric (serum total bilirubin level, 11.4 mg/dl). Generalized oozing from the peritoneum and the uterine incision was noted, and altered blood was present in the bowel. An upper gastrointestinal endoscopy failed to demonstrate a bleeding point. The patient was given a transfusion of 5 U of blood. The results of laboratory investigations are summarized in Table 1.

**Table 1:** Summary of laboratory results

	Postoperative day 2	Postoperative day 3	Postoperative day 4	Postoperative days 5 and 6	Postoperative day 10	Postoperative day 14
Hemoglobin (g/dl)	5	9	8.1	8.3	8.9	9.1
Platelet count (per liter)	60	53	40	35	118	190
Reticulocyte count (%)	4.5	–	5.8	–	–	–
Serum creatinine (mg/dl)	1.7	1.8	1.7	1.8	0.6	0.6
Blood urea nitrogen (mg/dl)	106.5	96	112	117	29.5	24
Serum potassium	3.8	3.9	5.3	5.1	3.6	3.7
SGOT (U/L)	79	84	104	111	88	77
SGPT (U/L)	47	77	97	103	80	73
ALP (U/L)	559	–	740	–	–	–
Serum bilirubin (mg/dl)						
Total	11.4					
Direct	7.6	13.6	15	15.7	5.8	4.3
Indirect	3.8					
Prothrombin time (S)	16	15	17.1	16	15	16
INR	1.7	1.7	1.5	1.5	1.6	1.7
CRP (mg/dl)	96	–	–	105		–
Serum total calcium (mmol/L)	1.8	–	–	–	2.0	–
Serum LDH (U/L)	–	–	1344	1490	310	–
Blood picture	–	–	–	Microangiopathic hemolytic anemia and suggestive of TTP	Recovery phase of microangiopathic hemolytic anemia	–
D-dimer (mg/dl)	–	–	–	2.76	1.8	
Blood culture	–	–	–	No growth	–	–
Upper gastrointestinal endoscopy	–	No specific bleeding point seen.	–	–	–	–
Brain CT scan	–	Normal	–	–	–	–
Brain MRI scan	–	Normal	–	–	–	–
Abdominal ultrasound scan	–	–	–	–	Liver is normal Mild ascites noted	–

*Abbreviations: SGOT* Aspartate aminotransferase, *SGPT* Alanine aminotransferase, *ALP* Alkaline phosphatase, *INR* International normalized ratio, *CRP* C-reactive protein, *LDH* Lactate dehydrogenase, *CT* Computed tomography, *MRI* Magnetic resonance imaging, *TTP* Thrombotic thrombocytopenic purpura

On the third postoperative day, the patient developed a focal seizure involving the right arm, but imaging of the brain failed to show a focal lesion. A diagnosis of TTP was made, and five cycles of plasmapheresis were completed. After plasmapheresis, her blood picture suggested the recovery phase of microangiopathic hemolytic anemia. Her hematological and biochemical parameters returned to normal, and she was discharged on the 16th postoperative day. At discharge, she had no complaints, and her blood pressure was 140/90 mmHg.

The patient was readmitted 12 hours following discharge with a complaint of acute-onset progressively worsening shortness of breath. She had features of acute left ventricular failure with a heart rate of 120 beats per minute, blood pressure of 160/100 mmHg, and arterial oxygen saturation of 90% while on oxygen. She was transferred to a coronary care unit, where she received aggressive treatment for heart failure. 2D echocardiography showed an ejection fraction of 45%, and a diagnosis of PPCM was made. Her B-type natriuretic peptide level was extremely elevated (31,400 U/L), and her troponin I titer was normal, confirming acute heart failure. She was started on bromocriptine, warfarin, and a heart failure regimen. At 6 weeks postpartum, her ejection fraction had improved to 60%, and she was normotensive.

## Discussion

TTP has a classic pentad of clinical features, including fever, thrombocytopenia, hemolytic anemia, neurological abnormalities, and renal failure [1]. In our patient, hemoperitoneum and hematemesis could be attributed to thrombocytopenia of TTP. Early plasmapheresis is the treatment of choice for TTP. Its effectiveness has been attributed to the removal of ADAMTS13 (a disintegrin and metalloproteinase with a thrombospondin type 1 motif, member 13) autoantibodies and replacement of ADAMTS13 activity [2, 3]. Disseminated intravascular coagulation (DIC) and transfusion-associated circulatory overload (TACO) can be other differential diagnoses. Even though prothrombin time prolongation and downward trend of platelet count were seen in the first few days in our patient, her D-dimer level was not high. Therefore, her presentation favors TTP rather than DIC as the possible diagnosis. TACO includes any four of the following occurring within 6 hours of a blood transfusion: acute respiratory distress, tachycardia, increased blood pressure, acute or worsening pulmonary edema, and evidence of a positive fluid balance [4]. Absence of these features in our patient excludes TACO.

Lactate dehydrogenase acts as a marker for the extent of hemolysis and response to plasmapheresis [5]. Reports in the literature show lower rates of relapse after splenectomy and also with the use of rituximab [6–8]. There is a significant risk of relapse in subsequent pregnancies [6]. Preconception counseling regarding potential risks in subsequent pregnancies is therefore important. Because TTP is rare, a high index of suspicion is required for rapid diagnosis and prompt treatment. Unexplained occurrence of thrombocytopenia and anemia should prompt consideration of the diagnosis and immediate evaluation of a peripheral blood smear.

Von Willebrand factor (VWF) is a large glycoprotein that circulates in plasma as a series of multimers. It plays a major role in primary hemostasis by inducing the formation of temporary platelet plugs at sites of vascular injury. Its activity is dependent on the sizes of the multimers, with ultralarge VWF multimers being biologically very potent [9, 10]. Dysfunctional VWF proteolysis resulting in the formation of VWF- and platelet-containing thrombi in the microcirculation of organs is implicated in the pathogenesis of TTP [9, 10]. The defective breakdown of VWF is attributed to a severely deficient activity of the VWF-cleaving protease ADAMTS13, a plasma metalloprotease synthesized in the liver, kidneys, and endothelium [9]. This protease rapidly degrades VWF-platelet strings by proteolytic cleavage of the VWF subunit, thereby regulating the size of the platelet thrombus [9, 10]. Congenital TTP occurs as a result of ADAMTS13 mutations, with the debut usually occurring in the first years of life, whereas acquired TTP is associated with autoantibodies against ADAMTS13 [9]. Many patients with TTP exhibit positive autoimmune reactions to other antigens, suggesting that defective immune regulation may contribute to the development of TTP [11]. Acquired TTP is considered a specific autoimmune disease characterized by antibodies, usually immunoglobulin G, directed against ADAMTS13 [6, 11]. These ultralarge VWF multimers cause spontaneous platelet aggregation in the microvasculature of the brain, kidney, and heart [6]. These account for the vascular episodes that are typical of TTP.

PPCM is characterized by left ventricular systolic dysfunction and heart failure. Its pathogenesis is not clear, but the role of autoimmunity in cardiovascular diseases has become one of the focal points in the field of cardiomyopathy and heart failure. The β_1_-adrenergic and M2-muscarinic receptors belong to the family of cardiac G protein-coupled receptors. Circulating autoantibodies against the second extracellular loop of β_1_-adrenergic receptors and M2-muscarinic receptors have been detected in a number of cardiovascular diseases characterized by heart failure, including idiopathic dilated cardiomyopathy and chronic Chagas heart disease [12, 13]. Patients with PPCM have been confirmed to be positive for autoantibodies against β_1_-adrenergic receptors (β_1_R-AABs) and autoantibodies against M2-muscarinic receptors (M_2_R-AABs). High titers of autoantibodies against selected cardiac tissue proteins have been found in the majority of women with PPCM [14]. An autoimmune mechanism is believed to participate in the pathogenesis of PPCM [15, 16].

A recent study has demonstrated the presence of β_1_R-AABs and M_2_R-AABs in patients with PPCM [15]. Positivity rates and serum titers of β_1_R-AABs and M_2_R-AABs were positively correlated with left ventricular dimensions and negatively correlated with the cardiac function of patients with PPCM [15]. The autoantibodies of cardiovascular receptors were independent risk factors for the onset of PPCM [15]. After 12 months of treatment for PPCM, the frequency and geometric mean titers of both β_1_R-AABs and M_2_R-AABs decreased significantly [15]. These have led to a proposal that autoantibodies against cardiovascular receptors may be involved in the pathogenesis of PPCM [15].

Effective management of PPCM reduces mortality and increases the number of women who fully recover their left ventricular function [17]. Outcomes for subsequent pregnancies are better in women who have fully recovered cardiac function after PPCM [18]. However, recurrence of cardiac failure is common in subsequent pregnancies [19]. We used bromocriptine, which has been shown to be effective in PPCM, to treat our patient [17]. There is one other case report in the literature describing cardiomyopathy occurring in pregnancy in association with TTP. This was reversed rapidly with combined plasma exchange and infusion [20].

## Conclusions

Our patient had two potentially lethal conditions of unknown etiology. Interestingly, the most plausible explanation for both conditions is an autoimmune mechanism.

## Abbreviations

ADAMTS13: A disintegrin and metalloproteinase with a thrombospondin type 1 motif, member 13)
β_1_R-AABs: Anti-β_1_-adrenergic receptor autoantibodies
DIC: Disseminated intravascular coagulation
M_2_R-AABs: Anti-M2-muscarinic receptor autoantibodies
PPCM: Peripartum cardiomyopathy
TACO: Transfusion-associated circulatory overload
TTP: Thrombotic thrombocytopenic purpura
VWF: von Willebrand factor

## References

[1] Gaddam S, Pablani L, Chainani V, Kavuda RR. Complete recovery of ischemic cardiomyopathy from thrombotic thrombocytopenic purpura. Clin Med Insights Cardiol. 2011;5:29–33. 10.4137/CMC.S6130 PMC3072209 21487455

[2] Rock GA, Shumak KH, Buskard NA, *et al*. Comparison of plasma exchange with plasma infusion in the treatment of thrombotic thrombocytopenic purpura. N Engl J Med. 1991;325:393–7. 10.1056/NEJM199108083250604 2062330

[3] Sadler JE, Moake JL, Miyata T, George JN. Recent advances in thrombotic thrombocytopenic purpura. In: Hematology Am Soc Hematol Educ Program; 2004. p. 407–23. 10.1182/asheducation-2004.1.407 15561695

[4] Blumberg N, Heal JM, Gettings KF, Phipps RP, Masel D, Refaai MA, *et al*. An association between decreased cardiopulmonary complications transfusion-related acute lung injury and transfusion-associated circulatory overload and implementation of universal leukoreduction of blood transfusions. Transfusion. 2010;50:2738–44. 10.1111/j.1537-2995.2010.02748.x PMC2944002 20561296

[5] James N, George MD. Thrombotic thrombocytopenic purpura. N Engl J Med. 2006;354:1927–35. 10.1056/NEJMcp053024 16672704

[6] Scully M, Hunt BJ, Benjamin S, *et al*. Guidelines on the diagnosis and management of thrombotic thrombocytopenic purpura and other thrombotic microangiopathies. Br J Haematol. 2012;158:323–35. 10.1111/j.1365-2141.2012.09167.x 22624596

[7] Crowther MA, Heddle N, Hayward CPM, *et al*. Splenectomy done during hematologic remission to prevent relapse in patients with thrombotic thrombocytopenic purpura. Ann Intern Med. 1996;125:294–6. 10.7326/0003-4819-125-4-199608150-00006 8678392

[8] Ono T, Mimuro J, Madoiwa S, *et al*. Severe secondary deficiency of von Willebrand factor-cleaving protease (ADAMTS13) in patients with sepsis-induced disseminated intravascular coagulation: its correlation with development of renal failure. Blood. 2006;107:528–34. 10.1182/blood-2005-03-1087 16189276

[9] Manea M, Karpman D. Molecular basis of ADAMTS13 dysfunction in thrombotic thrombocytopenic purpura. Pediatr Nephrol. 2009;24(3):447–58. 10.1007/s00467-008-0986-8 18807073

[10] Feys HB, Liu F, Dong N, Pareyn I, Vauterin S, *et al*. ADAMTS-13 plasma level determination uncovers antigen absence in acquired thrombotic thrombocytopenic purpura and ethnic differences. J Thromb Haemost. 2006;4(5):955–62. 10.1111/j.1538-7836.2006.01833.x 16689741

[11] Coppo P, Bengoufa D, Veyradier A, *et al*. Severe ADAMTS13 deficiency in adult idiopathic thrombotic microangiopathies defines a subset of patients characterized by various autoimmune manifestations, lower platelet count, and mild renal involvement. Medicine (Baltimore). 2004;83:233–44. 10.1097/01.md.0000133622.03370.07 15232311

[12] Fu LX, Magnusson Y, Bergh CH, Liljeqvist JA, Waagstein F, *et al*. Localization of a functional autoimmune epitope on the muscarinic acetylcholine receptor-2 in patients with idiopathic dilated cardiomyopathy. J Clin Invest. 1993;91:1964–8. 10.1172/JCI116416 PMC288192 7683693

[13] Sterin-Borda L, Gorelik G, Borda E. Chagasic IgG bingding with cardiac muscarinic cholinergic receptors modifies cholinergic-mediated cellular transmembrane signals. Clin Immunol Immunopathol. 1991;61:389–97. 10.1016/s0090-1229(05)80010-8 1657467

[14] Selle T, Renger I, Labidi S, Bultmann I, Hilfiker-Kleiner D. Reviewing peripartum cardiomyopathy: current state of knowledge. Future Cardiol. 2009;5:175–89. 10.2217/14796678.5.2.175 19371191

[15] Liu J, Wang Y, Chen M, Zhao W, Wang X, Wang H, Zhang Z, Zhang J, Xu L, Chen J, Yang X, Zhang L. The correlation between peripartum cardiomyopathy and autoantibodies against cardiovascular receptors. PLoS One. 2014;9(1):e86770. 10.1371/journal.pone.0086770 PMC3900643 24466231

[16] Ansari AA, Fett JD, Carraway RE, Mayne AE, Onlamoon N, Sundstrom JB. Autoimmune mechanisms as the basis for human peripartum cardiomyopathy. Clin Rev Allergy Immunol. 2002;23(3):301–24. 10.1385/CRIAI:23:3:301 12402414

[17] Sliwa K, Hilfiker-Kleiner D, Petrie MC, Mebazaa A. Current state of knowledge on aetiology, diagnosis, management, and therapy of peripartum cardiomyopathy: a position statement from the Heart Failure Association of the European Society of Cardiology Working Group on peripartum cardiomyopathy. Eur J Heart Fail. 2010;12(8):767–78. 10.1093/eurjhf/hfq120 20675664

[18] Sliwa K, Fett J, Elkayam U. Peripartum cardiomyopathy. Lancet. 2006;368(9536):687–93. 10.1016/S0140-6736(06)69253-2 16920474

[19] Murali S, Baldisseri MR. Peripartum cardiomyopathy. Crit Care Med. 2005;33(10 Suppl):S340–6. 10.1097/01.ccm.0000183500.47273.8e 16215357

[20] Cosmai EM, Puzis L, Tsai HM, Lian EC. Thrombocytopenic purpura and cardiomyopathy in pregnancy reversed by combined plasma exchange and infusion. Eur J Haematol. 2002;68(4):239–42. 10.1034/j.1600-0609.2002.01605.x 12071941

